# Association between composite dietary antioxidant index and Epstein–Barr virus infection in children aged 6–19 years in the United States: from the national health and nutrition examination survey 2007–2010

**DOI:** 10.3389/fnut.2024.1496410

**Published:** 2025-01-03

**Authors:** Wei Cheng, Yunfei Wang, Nan Ding, Rutao Xie

**Affiliations:** Surgery Department of Traditional Chinese Medicine, Longhua Hospital Affiliated to ShanghaiUniversity of Traditional Chinese Medicine, Shanghai, China

**Keywords:** children, composite dietary antioxidant index, Epstein–Barr virus infection, U-shaped, National Health and Nutrition Examination Survey

## Abstract

**Objective:**

Epstein-Barr virus (EBV) is a globally prevalent herpes virus associated with multiple diseases. Oxidative stress is closely related to EBV infection, latency, reactivation, and transformation. Antioxidant diet protects against EBV infection. Composite Dietary Antioxidant Index (CDAI), serving as a key measure of antioxidant intake, is a summary score of six dietary antioxidants, including vitamins A, C, and E, carotenoid, selenium, and zinc. Despite this, the association between CDAI and EBV infection remains uncertain.

**Methods:**

The aim of the study was to evaluate the association between CDAI and EBV infection using cross-sectional data from 3,318 children aged 6–19 years who participated in the American National Health and Nutrition Examination Survey between 2007 and 2010. Data on EBV results, CDAI, and several other essential variables were analyzed.

**Results:**

Compared with that of individuals in Q3 (−1.627–−0.2727) in the multivariate weighted logistic regression model with full adjustment for confounding variables, the adjusted odds ratio (OR) for CDAI and EBV infection in those in Q1 (−6.613 − −2.9157), Q2 (−2.9158–−1.626), Q4 (−0.2728–1.7601), and Q5 (1.7602–21.419) was 1.41 (95% CI: 1.01–1.96, *p* = 0.043), 1.10 (95% CI: 0.84–1.45, *p* = 0.447), 1.14 (95% CI: 0.86–1.51, *p* = 0.343), and 1.41 (95% CI: 1.01–1.98, *p* = 0.044), respectively. The association between CDAI and EBV infection showed a U-shaped curve (non-linear; *p* = 0.002). The OR of reducing EBV infection was 0.882 (95% CI: 0.792–0.982, *p* = 0.025) in participants with a CDAI of ≤ − 0.81. The OR of developing EBV infection was 1.055 (95% CI: 1.000–1.114, *p* = 0.050) in participants with a CDAI of > − 0.81.

**Conclusion:**

Our results indicated that the association between CDAI and EBV infection in U.S. adolescents follows a U-shaped curve, with an inflection point around –0.81.This suggests that an antioxidant-rich diet in some amount could help reduce the risk of EBV infection. Future prospective and experimental studies are needed to confirm causality and clarify the exact mechanism concerning antioxidant diets with EBV infection.

## 1 Introduction

The Epstein–Barr virus (EBV) is a gamma-herpesvirus with oncogenic properties that is associated with various neoplastic and autoimmune diseases. It is also linked to non-malignant diseases, including infectious mononucleosis, oral hairy leukoplakia, systemic lupus erythematosus, and multiple sclerosis (1). Primary infections typically occur early in life, especially during childhood (2). Humans are generally susceptible to EBV, and more than 95% of healthy adults are symptomatically infected with EBV worldwide (3). Young children are asymptomatic during the primary EBV infection, while adolescents and young adults often exhibit symptoms of infectious mononucleosis and other manifestations associated with primary EBV infections, which tend to persist lifelong (latency) by residing in memory B cells. EBV can reactivate and replicate when the immune system is weakened due to disease or immunosuppression, which may even lead to EBV-related cancers (4). Oxidative stress occurs when the production of reactive oxygen species exceeds the body’s ability to counteract them with antioxidants (5). It can also reactivate the EBV lytic cycle (6, 7). A study demonstrated that antioxidants protect against EBV lysis (8). Dietary adjustments may be effective in reducing EBV lysis by lowering the oxidative stress level within the body. Therefore, exploring an antioxidant-rich diet that may aid in the prevention or treatment of EBV infection is essential. The Composite Dietary Antioxidant Index (CDAI) is a reliable tool for evaluating the antioxidant properties of a diet. It is a summary score of six dietary antioxidants, including vitamins A, C, and E, carotenoids, selenium and zinc (9). An Italian study found that dietary antioxidant intake could protect against HPV infection and persistence, thereby reducing the risk of high-risk human papilloma virus infection (10). However, no studies have explored the link between CDAI and the risk of EBV infection. To address this knowledge gap, we evaluated the relationship between CDAI and EBV in adolescents using data from the National Health and Nutrition Examination Survey (NHANES). We investigated how dietary guidance might help reduce the risk of EBV infection. Additionally, we assessed the dose–response relationship.

## 2 Materials and methods

### 2.1 Data source and study design

The National Health and Nutrition Examination Surveys (NHANES) is an ongoing nationwide survey in the United States that assesses health and nutrition, conducted by the Centers for Disease Control and Prevention. The NHANES compiles large volumes of data pertaining to the demographic characteristics, healthy behaviors, and nutrition of individuals in the US (11). Researchers can access NHANES datasets and corresponding statistics on the official NHANES website.^1^ The NHANES is authorized by the National Center for Health Statistics Ethics Review Committee, and all participants provide written informed consent before participating. Additionally, the secondary analysis does not require further Institutional Review Board approval (12). Before participating, parents or guardians provided written informed consent, and minors aged 7–15 years also provided additional signed assent (13).

**Figure 1:**
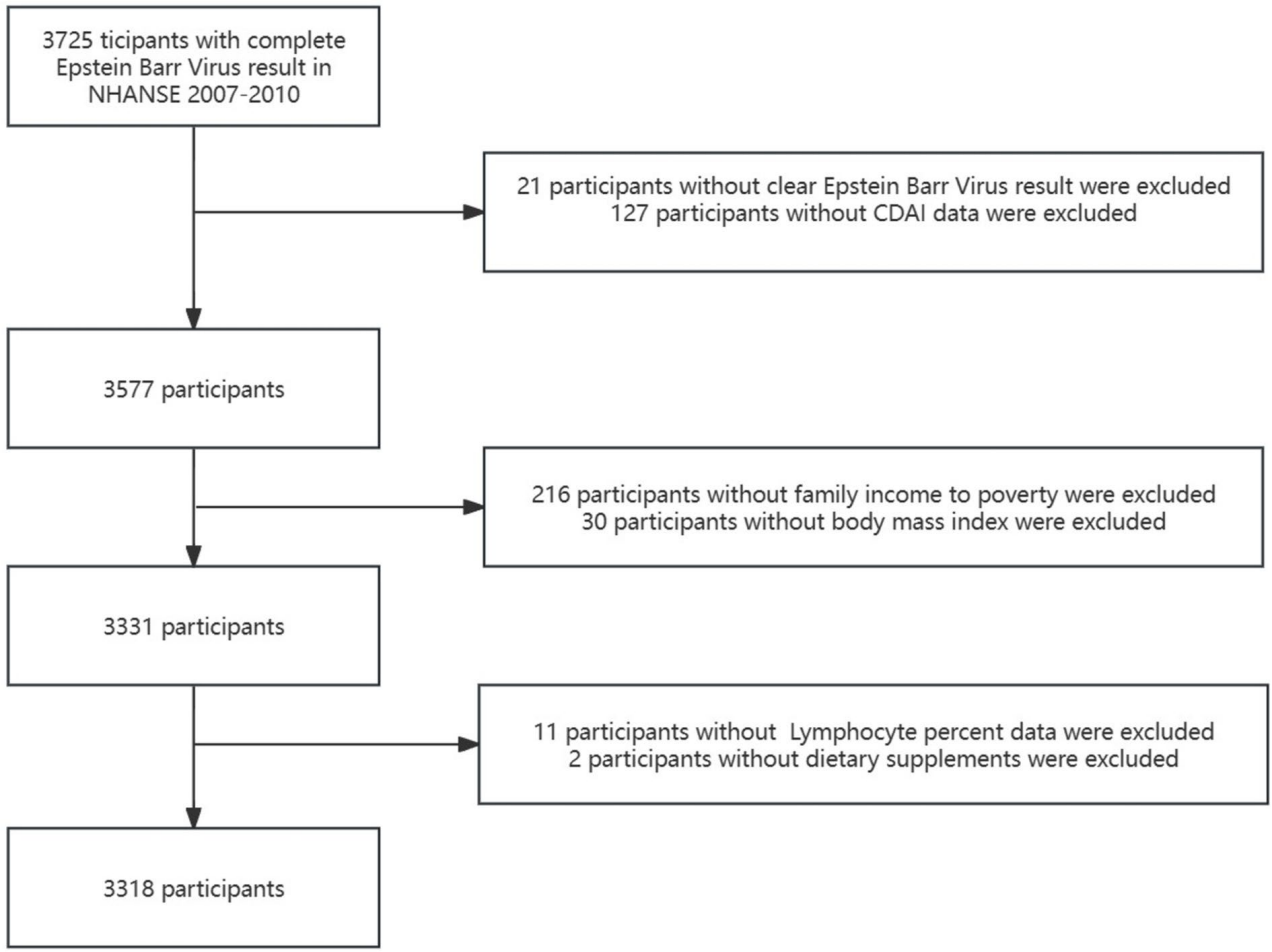
The study’s flow diagram. CDAI, Composite Dietary Antioxidant Index.

This cross-sectional study used NHANES data from 2007 to 2010 obtained from the Centers for Disease Control and Prevention [National Center for Health Statistics NHANES Survey Methods and Analytic Guidelines. [(accessed on 1 July 2024)]; Available online: https://wwwn.cdc.gov/nchs/nhanes/AnalyticGuidelines.aspx]. A total of 3,725 participants with complete Epstein–Barr virus data constituted the study sample. We excluded participants with an equivocal and uncertain Epstein–Barr virus result (*n* = 21), with the missing data of Composite Dietary Antioxidant Index (*n* = 127), with the missing value of covariates [ratio of family income to poverty (PIR) (*n* = 216), body mass index (BMI) (*n* =30), lymphocyte percentage (*n* = 11) and dietary supplement (*n* = 2)]. Finally, a total of 3,318 participants were included in the present study. These participants represented a weighted population of 52,177,715 individual’s non-institutionalized children in the US. Figure 1 illustrates the selection process employed in the study.

### 2.2 Measurement of dietary antioxidant intake

We obtained detailed dietary intake information from NHANES participants between 2007 and 2010 through dietary interviews followed by EBV antibody testing for children aged 6–19 years at a Mobile Examination Center (MEC). The data recorded via dietary recall were used to estimate the types and amounts of foods and beverages consumed during the 24-h (midnight to midnight) period before the interview and to estimate the intake of energy, nutrients, and other food components (14). According to previous literature (15, 16), We evaluated data from the first 24-h recall interviews using a modified version of the CDAI developed by Wright et al. (17). The assessment included vitamins A, C, and E, carotenoid along with minerals from food sources such as selenium, and zinc. The CDAI equation is as follows:


$$\mathrm{CDAI}=\sum_{i=1}^{n=6}\left(\mathrm{Individual Intake}-\mathrm{Mean}\right)/\mathrm{standard deviation}$$

### 2.3 Epstein–Barr virus antibody testing

The data related to EBV outcomes in this study were sourced from NHANES laboratory data. EBV antibody testing was conducted among children aged 6–19 years, and EBV Anti-capsid Immunoglobulin (VCA IgG) antibody levels were measured using a commercial enzyme immunoassay kit (Diamedix, Miami, FL). All quality assurance and quality control (QA/QC) procedures recommended by the manufacturer were strictly adhered to. The sensitivity of the assay is 96.6% and the specificity is 97.7%. A detailed description of laboratory methodology is available on the NCHS website (18).^2^ Enzyme Immunoassay (EIA) indices were calculated by hand using the formulas in the kit package insert to identify EBV infection. EIA indices of <0.9 were considered negative, EIA indices of ≥1.10 were considered positive and EIA index ranging from 0.90 to 1.09 were considered equivocal.

### 2.4 Covariates

We aimed to minimize potential confounding biases in the analyses by selecting covariates based on previous research (14) (including age, sex, race, ratio of family income to poverty(PIR), Body Mass Index(BMI), dietary supplement taken, lymphocyte percentage, monocyte percentage, segmented neutrophils percentage, lymphocyte count, and monocyte count). Age groups were classified as ≤10 and > 10 years. Race was categorized as Mexican American, other Hispanic, non-Hispanic White, non-Hispanic Black, and Other. BMI was defined as weight/(height)^2^ kg/m^2^. It is categorized into three groups: underweight (BMI ≤ 18.5 kg/m^2^), normal weight (18.5 kg/m^2^ < BMI ≤ 25 kg/m^2^), and overweight (BMI > 25 kg/m^2^). The PIR was also divided into three categories: low income (PIR ≤ 1), middle income (1 < PIR ≤ 4), and high income (PIR > 4). A personal interview was conducted to obtain data on the use of dietary supplements during a 30-day period before the survey date. Dietary supplement values were categorized as ≤4 (few) and > 4 (more). Some of the parameters indicating systemic inflammation included lymphocyte percent (%), monocyte percent (%), segmented neutrophils percent (%), lymphocyte number (1,000 cells/μL), and monocyte number (1,000 cells/μL). Blood samples from all participants were analyzed for complete blood count (CBC) using the Beckman Coulter HMX instrument at the NHANES MEC. The methods used to derive CBC parameters were based on the Beckman Coulter method of counting and sizing.

### 2.5 Statistical analyses

Participants were divided into five groups according to their CDAI quintiles. The baseline characteristics of the groups were compared. Continuous variables were presented as mean (standard deviation, SD) or median (interquartile range, IQR) whereas categorical variables were presented as frequencies and proportions. To compare differences among the CDAI quintiles, we used either a one-way analysis of variance for normally distributed data or a Kruskal-Wallis H test for skewed data. We utilized the chi-square test, or Fisher’s exact test when appropriate, to compare the characteristics of participants across the five groups. Individual sample weights were determined using specific sample weights for Epstein–Barr Virus subsample (WTSSE2Y) records, and WTSSEB2Y/2 was applied based on the chosen cycle to extrapolate results to the entire non-institutionalized US adolescent population.

We employed multivariate weighted logistic regression analyses to calculate the odds ratios (ORs) and 95% confidence intervals (95% CIs) for the relationship between CDAI and EBV infection, including an unadjusted and two adjusted models. The middle group of CDAI (Q3) was used as the reference group among the five CDAI groups. Model 1 was adjusted for age, sex, race, PIR, and BMI, while Model 2 was adjusted for dietary supplements, lymphocyte percentage, monocyte percentage, segmented neutrophil percentage, lymphocyte number, and monocyte number. The potential non-linear relationship between CDAI and EBV infection was examined using smooth curve fitting (restricted cubic splines) and weighted generalized additive model after adjusting for the variables in Model 2. Subgroup analyses based on age, sex, BMI, and PIR were performed to assess the reciprocal effect of CDAI-EBV infection association across various subgroups. We used a two-piecewise logistic model and recursive algorithm to analyze the inflection point between CDAI and EBV infection after adjusting for variables in Model 2. The likelihood-ratio test was used to determine the inflection points, and all the analyses were performed using the statistical software packages R^3^ and the Free Statistics software (version 1.9.2; Beijing Free Clinical Medical Technology Co., Ltd., Beijing, China) (19), with statistical significance set at a two-tailed *p*-value of <0.05.

## 3 Results

### 3.1 Baseline characteristics

A total of 3,318 adolescents with complete data were included in the analyses. The sample was weighted to represent the entire US adolescent population (52.1 million). Table 1 illustrates the baseline characteristics of all participants according to their CDAI quintile. All participants were aged 6–19 years, and 64.09% of participants were infected with EBV. These participants included 51.26% male individuals, 48.74% female individuals, and 60.30% non-Hispanic White individuals. Sex, age, EBV result, lymphocyte percent, segmented neutrophil percent, and lymphocyte number in the various quintile groups were significantly different (*p* < 0.05). The highest CDAI quintile group was predominantly male (*p* = 0.0466) and older (*p* < 0.0001), with higher EBV infection rates (*p* = 0.0057), lower lymphocyte percent (*p* = 0.0047), and lymphocyte number (*p* = 0.0016), and higher segmented neutrophil percent (*p* = 0.0021). In contrast, the middle CDAI quintile group had higher lymphocyte percent, and lymphocyte number but lower segmented neutrophil percent.

**Table 1:** Demographic characteristics stratified by CDAI quintile.

Variables	Dietary antioxidant index						*p*
	Overall	Q1(−6.613–-2.9157)	Q2(−2.9158–-1.626)	Q3(−1.627–-0.2727)	Q4(−0.2728–1.7601)	Q5(1.7602–21.419)	
*N*	52177715.25	10285013.68	9995204.81	10619665.00	10343372.45	10934459.31	
Age (year), *n* (%)							<0.0001^*^
≤10	14208684.45 (27.23)	2479339.17 (24.11)	3580286.43 (35.82)	3498673.95 (32.95)	2640569.97 (25.53)	2009814.93 (18.38)	
>10	37969030.80 (72.77)	7805674.52 (75.89)	6414918.37 (64.18)	7120991.05 (67.05)	7702802.48 (74.47)	8924644.38 (81.62)	
Sex, *n* (%)							0.0466^*^
Male	26746690.39 (51.26)	4674958.48 (45.45)	5226652.52 (52.29)	6089838.66 (57.34)	5144401.76 (49.74)	5610838.98 (51.31)	
Female	25431024.86 (48.74)	5610055.21 (54.55)	4768552.29 (47.71)	4529826.34 (42.66)	5198970.69 (50.26)	5323620.33 (48.69)	
Race, *n* (%)							0.2800
Mexican American	6793434.79 (13.02)	1436922.89 (13.97)	1404979.11 (14.06)	1432657.84 (13.49)	1385079.37 (13.39)	1133795.59 (10.37)	
Other Hispanic	3087759.07 (5.92)	570815.51 (5.55)	590778.74 (5.91)	579646.79 (5.46)	666527.65 (6.44)	679990.38 (6.22)	
Non-Hispanic White	31465072.06 (60.30)	5863781.62 (57.01)	6052086.01 (60.55)	6637079.80 (62.50)	6257785.41 (60.50)	6654339.20 (60.86)	
Non-Hispanic Black	7470217.80 (14.32)	1582644.03 (15.39)	1432471.14 (14.33)	1418009.11 (13.35)	1495789.60 (14.46)	1541303.93 (14.10)	
Other race	3361231.53 (6.44)	830849.63 (8.08)	514889.81 (5.15)	552271.46 (5.20)	538190.41 (5.20)	925030.22 (8.46)	
Body mass index (kg/m2) (%)							0.0584
≤18.5	17704588.69 (33.93)	3106531.94 (30.20)	3917198.58 (39.19)	3850929.96 (36.26)	3571742.64 (34.53)	3258185.57 (29.80)	
18.5–25	23003430.75 (44.09)	4424207.35 (43.02)	3916386.06 (39.18)	4752073.43 (44.75)	4590822.41 (44.38)	5319941.49 (48.65)	
>25	11469695.81 (21.98)	2754274.39 (26.78)	2161620.16 (21.63)	2016661.61 (18.99)	2180807.40 (21.08)	2356332.25 (21.55)	
Ratio of family income to poverty, *n* (%)							0.0749
≤1	12168466.20 (23.32)	2691387.86 (26.17)	2382600.58 (23.84)	2241213.04 (21.10)	2155426.38 (20.84)	2697838.34 (24.67)	
1–4	25985312.19 (49.80)	5436251.13 (52.86)	5048021.68 (50.50)	5327031.54 (50.16)	4900087.12 (47.37)	5273920.72 (48.23)	
>4	14023936.87 (26.88)	2157374.69 (20.98)	2564582.55 (25.66)	3051420.43 (28.73)	3287858.95 (31.79)	2962700.25 (27.10)	
Dietary supplements taken, *n* (%)							0.6331
0–4	51602317.52 (98.90)	10229266.77 (99.46)	9867719.27 (98.72)	10510491.62 (98.97)	10161784.00 (98.24)	10833055.86 (99.07)	
>4	575397.73 (1.10)	55746.91 (0.54)	127485.54 (1.28)	109173.38 (1.03)	181588.45 (1.76)	101403.45 (0.93)	
Epstein Barr Virus result, *n* (%)							0.0057^*^
Positive	33442589.25 (64.09)	7176391.65 (69.78)	6116124.27 (61.19)	6231041.61 (58.67)	6431527.94 (62.18)	7487503.77 (68.48)	
Negative	18735126.00 (35.91)	3108622.03 (30.22)	3879080.53 (38.81)	4388623.39 (41.33)	3911844.51 (37.82)	3446955.54 (31.52)	
Lymphocytepercent [median (IQR)]	35.300 [29.500, 41.800]	34.700 [29.900, 41.179]	36.400 [29.028, 42.807]	36.459 [30.300, 42.700]	36.400 [30.800, 42.504]	33.977 [28.000, 39.900]	0.0047^*^
Monocyte percent [median (IQR)]	8.200 [6.900, 9.700]	8.300 [6.900, 9.600]	8.076 [6.900, 9.900]	8.200 [7.100, 9.600]	8.200 [6.900, 9.700]	8.100 [6.800, 9.600]	0.7819
Segmented neutrophils percent [median (IQR)]	52.000 [45.000, 58.700]	52.700 [45.600, 58.400]	50.677 [43.600, 58.722]	50.812 [44.300, 58.600]	51.300 [44.400, 57.400]	54.106 [46.844, 60.300]	0.0021^*^
Lymphocytenumber [median (IQR)]	2.300 [1.900, 2.800]	2.300 [1.900, 2.800]	2.300 [1.900, 2.800]	2.400 [2.000, 3.000]	2.400 [2.000, 2.800]	2.200 [1.800, 2.700]	0.0016^*^
Monocyte number [median (IQR)]	0.600 [0.400, 0.700]	0.500 [0.500, 0.700]	0.500 [0.400, 0.700]	0.600 [0.500, 0.700]	0.600 [0.400, 0.700]	0.500 [0.414, 0.700]	0.1557

Q, quintiles; IQR, interquartile range; Q1:the first quintile, Q2:the second quintile, Q3:the third quintile, Q4:the fourth quintile, Q5:the fifth quintile. CDAI quintile range: quintile 1:-6.613−-2.9157; quintile 2: −2.9158−-1.626; quintile 3: −1.627–-0.2727; quintile 4:-0.2728–1.7601;quintile 5:1.7602–21.419.

### 3.2 Association between CDAI and EBV infection

**Figure 2:**
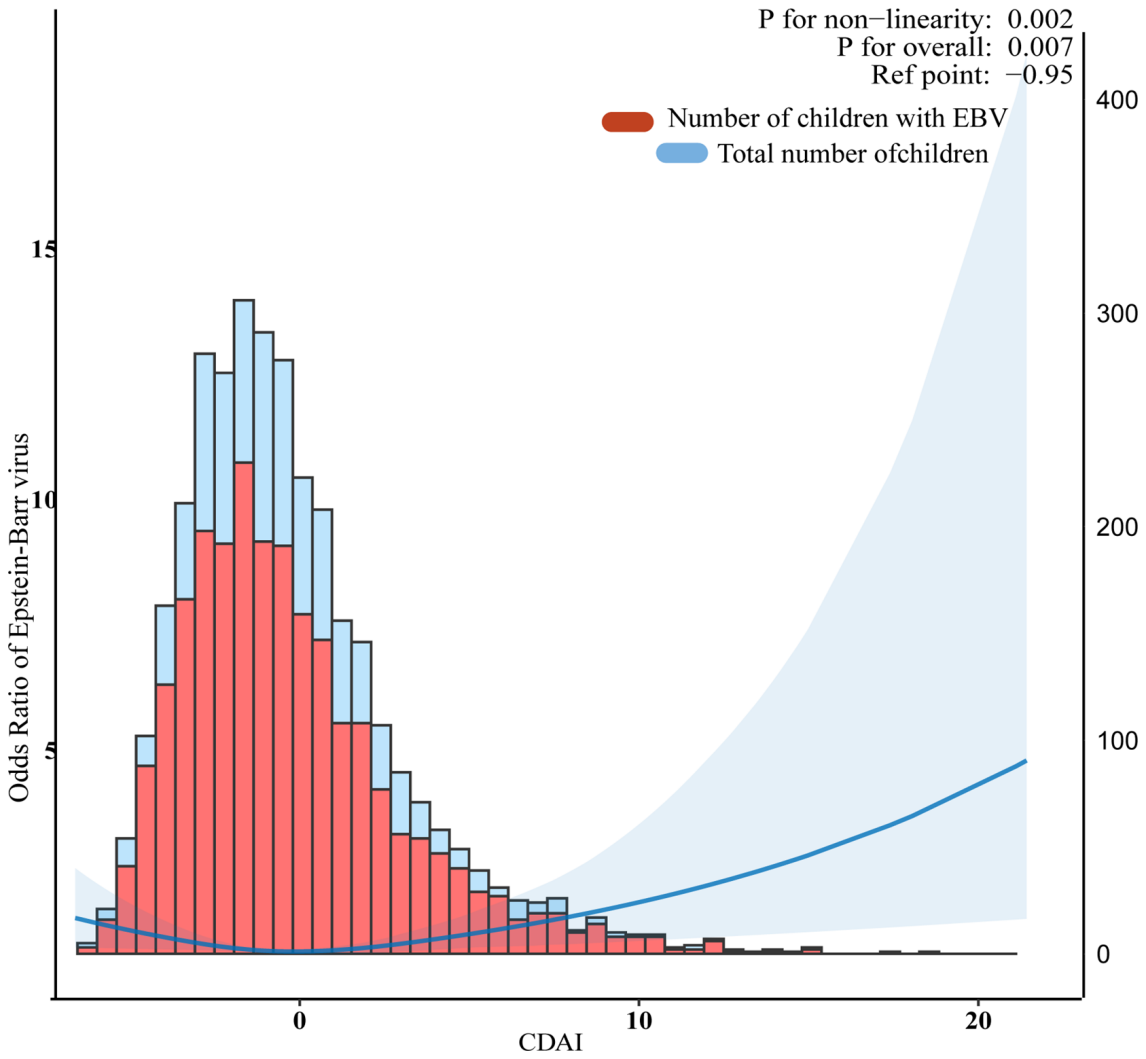
Association between composite dietary antioxidant index and Epstein Barr Virus infection. The blue line represents Odds Ratio. The blue area represents the 95% confidence interval of the fit. They were adjusted for sociodemographic (age, sex, race, ratio of family income to poverty), body mass index, dietary supplements taken, lymphocyte percent, monocyte percent, segmented neutrophils percent, lymphocyte number, and monocyte number.

In multivariate weighted logistic regression analyses, after adjusting for potential confounders (model 2), compared with individuals in the middle quintile (Q3:-1.627–-0.2727), the adjusted OR for CDAI and EBV infection in the lowest quintile (Q1:-6.613–-2.9157), the second quintile (Q2:-2.9158–-1.626), the fourth quintile (Q4:-0.2728–1.7601), and the fifth quintile (Q5:1.7602–21.419) was 1.41 (95% CI: 1.01–1.96, *p* = 0.043), 1.10 (95% CI: 0.84–1.45, *p* = 0.447), 1.14 (95% CI: 0.86-1.51, *p* = 0.343), and 1.41 (95% CI: 1.01–1.98, *p* = 0.044), respectively (Table 2). Accordingly, the association between CDAI and EBV infection exhibited a U-shaped association (nonlinear, *p* = 0.002) in restricted cubic spline (RCS) (Figure 2). In the threshold analysis, participants with a CDAI of ≤ −0.81 had an OR of 0.882 (95% CI: 0.792-0.982, p = 0.025) for developing EBV infection. In contrast, those with a CDAI of > −0.81 had an OR of 1.055 (95% CI: 1.000–1.114, *p* = 0.050; Table 3). This finding suggests that in participants with a CDAI of ≤ −0.81, the risk of EBV infection decreases by 11.8% for each unit increase in CDAI. In contrast, for those with a CDAI of > −0.81, the risk increases by 5.5% for each unit increase in CDAI.

**Table 2:** Association between composite dietary antioxidant index. and Epstein Barr Virus infection.

Quintiles	OR (95% CI)					
	Crude	*p*- value	Model 1	*p*-Value	Model 2	*p*- value
Dietary antioxidant index						
Q1 (−6.613- -2.9157)	1.63 (1.19-2.22)	0.003	1.40 (1.03-1.92)	0.034	1.41 (1.01-1.96)	0.043
Q2 (−2.9158- -1.626)	1.11 (0.85-1.46)	0.435	1.09 (0.84-1.41)	0.489	1.10 (0.84-1.45)	0.447
Q3 (−1.627--0.2727)	1 (Ref)		1 (Ref)		1 (Ref)	
Q4 (−0.2728–1.7601)	1.16 (0.88-1.52)	0.283	1.12 (0.86-1.47)	0.375	1.14 (0.86-1.51)	0.343
Q5 (1.7602–21.419)	1.53 (1.10-2.14)	0.014	1.41 (1.02-1.93)	0.037	1.41 (1.01-1.98)	0.044

Q, quintiles; OR, odds ratio; CI, confidence interval; Model 1: Age, sex, race, ratio of family income to poverty, and body mass index were adjusted. Model 2: Dietary supplements, lymphocyte percentage, monocyte percentage, segmented neutrophil percentage, lymphocyte number, and monocyte number were adjusted based on Model 1.

**Table 3:** Threshold effect analysis of the relationship of composite dietary antioxidant index with Epstein Barr Virus infection.

**Composite dietary antioxidant index**	**Adjusted model**	
	**OR (95% Cl)**	***p*- value**
≤ − 0.81	0.882 (0.792-0.982)	0.025
> − 0.81	1.055 (1.000-1.114)	0.050
Log-likelihood ratio test		0.011

OR, odds ratio; CI, confidence interval; adjusted for sociodemographic factors (age, sex, race, ratio of family income to poverty), body mass index, dietary supplements taken, lymphocyte percentage, monocyte percentage, segmented neutrophil percentage, lymphocyte number, and monocyte number.

### 3.3 Stratified analysis based on additional variables

**Figure 3:**
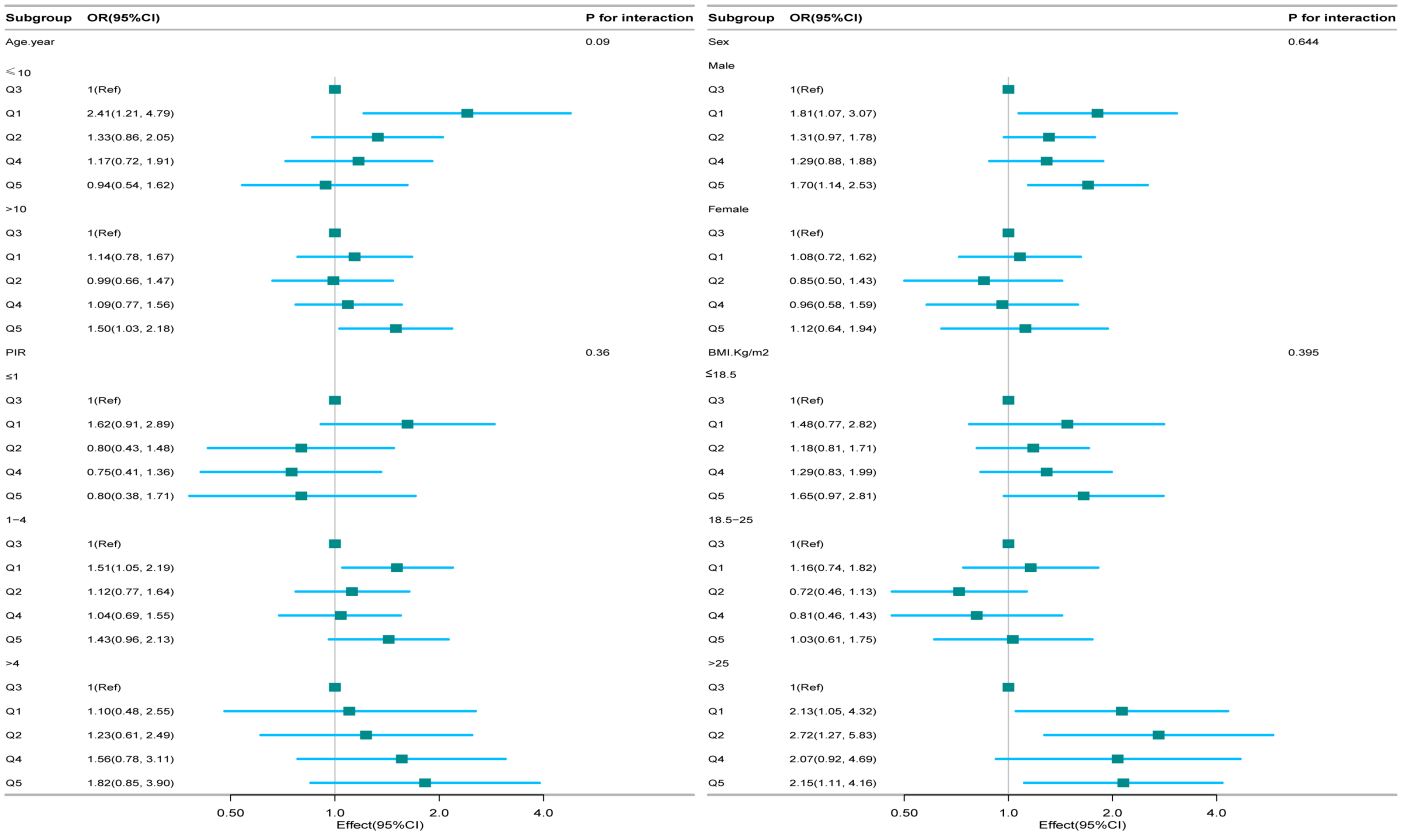
Subgroup analysis of the relationship between composite dietary antioxidant index and Epstein Barr Virus infection. Based on these features, except for the stratification component, each stratification factor was adjusted for all other variables (age, sex, race, ratio of family income to poverty), body mass index, dietary supplements taken, lymphocyte percentage, monocyte percentage, segmented neutrophil percentage, lymphocyte number, and monocyte number.

The analyses showed no significant association between CDAI and EBV infection across the subgroups stratified by sex, age, BMI, and PIR (Figure 3).

## 4 Discussion

The prevalence of EBV infection was estimated to be 64.09% among children aged 6–19 years in this cross-sectional study, which is lower than the global prevalence (>90%). This study introduces a new index of CDAI, which differs from previous studies. The CDAI is a dietary antioxidant index developed by Wright et al. to assess the comprehensive intake of antioxidant nutrients in the diet. Our findings reveal a U-shaped association between CDAI and the risk of EBV infection, with an inflection point of almost −0.81. Moreover, the association persisted even after adjusting for other covariates, indicating that CDAI is a protective factor for the development of EBV infection in individuals with a CDAI of ≤ − 0.81 and a risk factor for those with a CDAI of > − 0.81. These findings have important implications for managing EBV infection. To the best of our knowledge, this is the first large-sample study to explore the association between comprehensive antioxidant diet and EBV infection.

Some studies have confirmed that antioxidant therapy can reduce oxidant tissue damage associated with the response to viral infections (20). Antioxidants might exert an inhibitory effect on viral replication by modulating cytokines and gene expression (21). There are still no specific drugs to prevent or treat EBV infections, so preventing or treating related infections through an antioxidant diet may be a solution. Studies have confirmed that some antioxidants can inhibit EBV infection, latency, and reactivation (22). Tsushima et al. found that most carotenoids exhibit inhibitory EBV activation activity (23). Chang Guo et al. found that the antioxidant vitamin E can inhibit the EBV transformation in human B cells induced by H2O2 or cyclosporin A (CsA) (24). Chuang Chun et al. found that sulforaphane, an antioxidant, effectively inhibited EBV reactivation (25). Amor et al. found that olive extracts with antioxidant activity reduced the EBV lytic cycle (8). Although various studies have confirmed that individual antioxidants inhibit EBV activity, research on the link between an antioxidant diet and EBV infection is still limited. Nina found an inverse correlation between EBV VCA IgM and vitamin C in plasma. In addition, high-dose intravenous vitamin C therapy has a positive effect on the reduction of EBV antibody levels (26). This is partially consistent with our conclusion that CDAI above a certain range is inversely associated with EBV infection rate. This study deepens the analysis to assess whether dietary antioxidant intake is associated with EBV infection. Vitamin C has pro-oxidant and antioxidant properties with no negative effects. A study has shown that it can also have deleterious effects through the production of highly toxic ROS (27). Recommended intake of vitamin C should vary according to genetic background, quality of diet and extent of disease (28). Studies have shown that high doses of selenium elevate cellular ROS, which induces oxidative stress (29). High doses of selenium can lead to decreased immune function and impair endothelial function (30). Sun et al. found that the non-linear dose-response relationship between selenium status and health is a U-shaped, individuals with low baseline selenium levels may benefit from supplementation, whereas those with acceptable or high selenium levels may experience detrimental effects (31). This finding is consistent with our results. Suma et al. confirmed that high-dose zinc gluconate supplements did not reduce syndrome corona virus 2 symptoms. Zinc supplementation above the recommended doses may exert harmful side effects like medication absorption decrease (32).This finding may explain the association of high doses of antioxidants with rising EBV infections after the threshold was exceeded. Therefore, this study fills the literature gap between factors affecting EBV infection. In our study, higher dietary antioxidant intake was associated with lower EBV infection rates before the threshold was exceeded, and higher dietary antioxidant intake was associated with higher EBV infection rates after the threshold was exceeded. Nevertheless, our findings currently lack supporting evidence, prompting the need for large prospective cohort studies for further validation.

Antioxidant defense is a critical mechanism by which host cells defend themselves against pathogenic microorganisms. EBV infection induces oxidative stress during the lytic, latent, and reactivation stages (33). A recent study showed that EBV infection can directly affect the oxidative profile of *in vitro* cultivated human cells and establish oxidative stress, which can play a key role in viral transformation (34). Wang et al. found that the mitochondria in cell lines with EBV type 3 latency produce higher levels of mitochondrial reactive oxygen. EBV-triggered reactive oxygen production activates the Keap1-NRF2 pathway which is the master antioxidant defense mechanism in EBV-transformed cells. An imbalance between oxidative stress and antioxidant defense can influence cell proliferation and the reactivation of EBV (34). Hannah et al. used reactive oxygen species (ROS) production to stimulate the EBV lytic cascade to treat EBV-associated tumors (35). Vitamin C is an important cofactor in the immune system for scavenging reactive oxygen species, and its antioxidant properties help to protect cells and reduce oxidative damage during redox reactions (36). Vitamin E improves cell proliferation and immune function against viral infections (37). The un-chelated free form of Zinc have the antiviral effects (38). Low selenium concentrations are associated with a higher chance of viral infection. Selenium can increase T-cell counts and facilitate innate and adaptive immunity. Selenium has potent antioxidant properties, and supplementing selenium can improve the systemic redox state (36, 39). Although several analyses have shown the inhibitory effect of antioxidants on viral infections through antioxidant activity and enhancement of immune cell functions, further research is needed to understand how dietary antioxidants affect EBV at the biological level.

This study is notable as the first large-scale investigation linking CDAI with EBV infection in US adolescents. Our findings complement the literature on the association between dietary antioxidants and viral infection. Despite its strengths, the study has several limitations. First, as a cross-sectional study, it cannot establish causal relationships between CDAI and EBV infection. Second, dietary data were collected using 24-h recall interviews, which may not accurately reflect participants’ daily eating habits. Third, the study was limited by the availability of EBV tests in the database, which were only relevant for children aged 6 to 19 years, thus limiting the generalizability of the results to other populations. Finally, there may be additional confounding factors which may have influenced the results not being taken into consideration due to missing data.

## 5 Conclusion

The analysis of a nationally representative group of US adolescents revealed that CDAI was significantly and negatively correlated with EBV infection in individuals with a CDAI of ≤ − 0.81 and positively correlated in those with a CDAI of > − 0.81. This suggests that an antioxidant-rich diet in some amount could help reduce the risk of EBV infection. Therefore, promoting an antioxidant-rich diet may be an important prevention strategy for EBV infection. Future prospective and experimental studies are needed to confirm causality and clarify the exact mechanism concerning antioxidant diets with EBV infection.

## Data Availability

The original contributions presented in the study are included in the article/supplementary material, further inquiries can be directed to the corresponding author.

## Footnotes

^1^ www.cdc.gov/nchs/nhanes/

^2^ https://wwwn.cdc.gov/Nchs/Nhanes/2003-2004/SSEBV_C.htm

^3^http://www.R-project.org, The R Foundation
